# Rare Case of 6 cm Right Atrial Myxoma in Patient with Synchronous Endometrial Adenocarcinoma

**DOI:** 10.1155/2021/4657117

**Published:** 2021-10-11

**Authors:** Mohammad Altujjar, Feras Zaiem, Erin Sheehan, Willy Gan, Mohammed Mhanna, Waleed Khokher, Ikram Ullah, Mohammad Tokir Mujtaba

**Affiliations:** ^1^Dartmouth Hitchcock Medical Center, Lebanon, USA; ^2^Wayne State University, Detroit, USA; ^3^Indiana University, Indianapolis, Indiana, USA; ^4^University of Toledo, Toledo, USA

## Abstract

Primary cardiac tumors are extremely rare. Cardiac myxomas most frequently appear in the left atrium. In this article, we present a case of an asymptomatic 6 cm right atrial mass in a patient undergoing staging for endometrial cancer. The mass was resected, and final pathology was consistent with cardiac myxoma.

## 1. Case Presentation

### 1.1. History of Presentation

A 55-year-old female presented to the hospital with vaginal bleeding. Diagnostic hysteroscopy with dilation and curettage showed endometrioid endometrial adenocarcinoma. During staging, a CT chest revealed a 6 cm right heart atrial mass. The patient denied chest pain, shortness of breath, or palpation. Physical exam and auscultation of the heart and lung were unremarkable. Complete blood count and basic metabolic profile were within normal limits.

*Past medical history:* essential hypertension and hyperthyroidism

*Differential diagnosis:* benign cardiac tumor, malignant cardiac tumor, intracardiac thrombus, valvular vegetation, and pericardial cyst

### 1.2. Investigations

**Figure 1:**
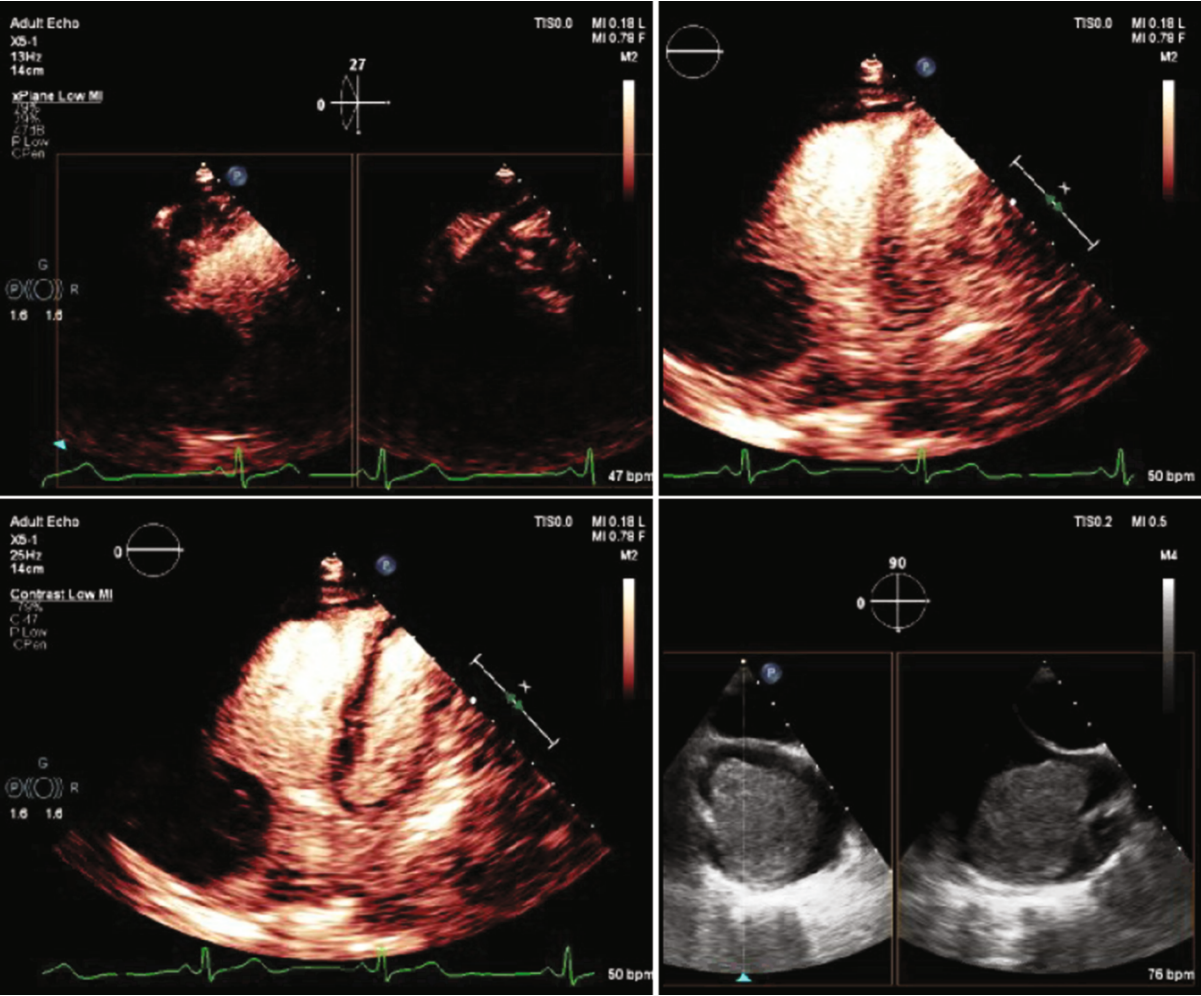
Transesophageal echocardiography showing echogenic right atrial mass with a possible noted stalk.

**Figure 2:**
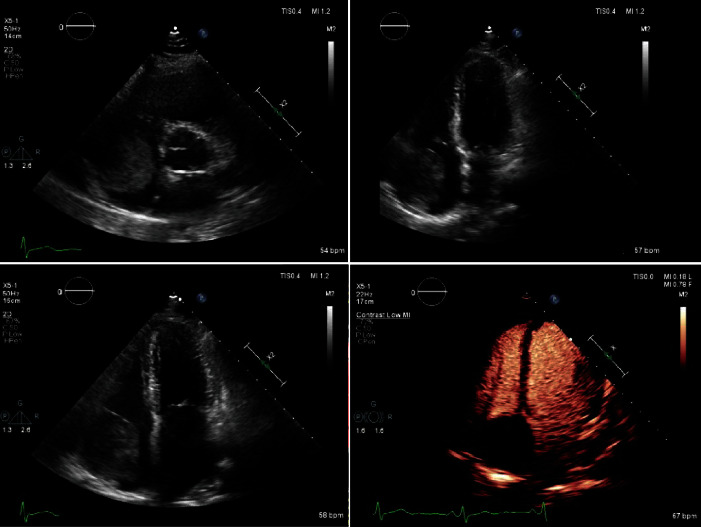
Transthoracic echocardiogram (TEE) view of the mass. Myocardial contrast echo findings suggest minimal to no vascularity within the mass.

A transthoracic echocardiogram (TTE) confirmed the presence of a large round echogenic right atrial mass with a possible stalk (Figure 1). Transesophageal echocardiogram (TEE) measured the echogenic mass at 5.5 × 4.6 cm, originating from a tiny stalk over the right atrium appendage (Figure 2). The patient underwent right and left heart catheterization to assess for possible coronary artery disease and was found to have no significant coronary artery disease. Right atrial angiogram demonstrated a large and immobile-appearing mass attached to the wall of the right atrium.

### 1.3. Management

**Figure 3:**
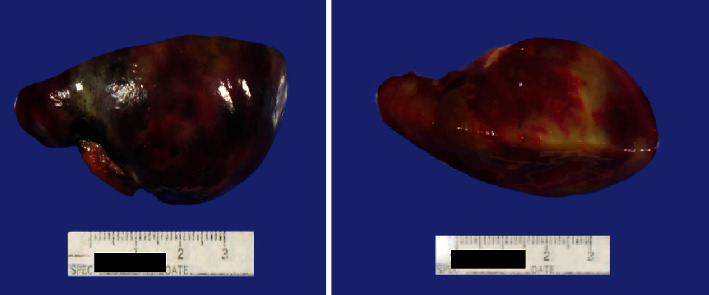
The resected tumor showed a 6 × 6 × 3.5 cm oval-shaped mass with a small lobulated stalk.

**Figure 4:**
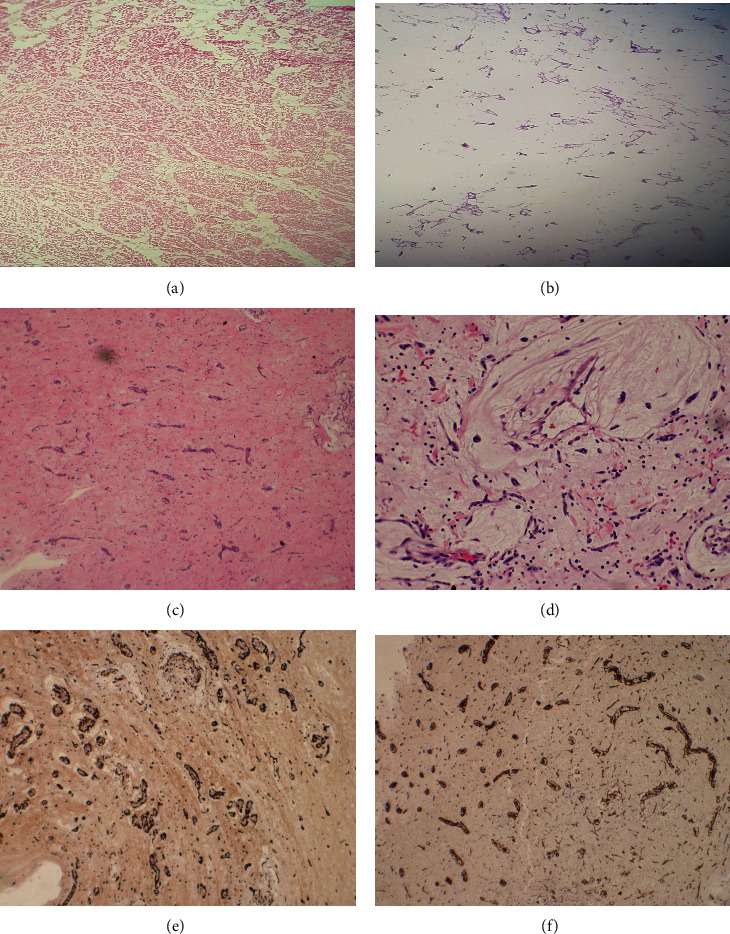
The intraoperative frozen section showed unremarkable skeletal heart muscle with abundant myxoid stroma (a, b). The hematoxylin and eosin histology sections (c, d) showed no cytologic atypia or necrosis. The lesional cells are immunoreactive for calretinin (e) and CD34 immunostains (f).

The patient underwent surgery and excision of the mass. The resected mass measured 6 × 6 × 3.5 with a small lobulated stalk measuring 2 × 2 cm (Figure 3). Intraoperative section was consistent with a benign lesion (Figures 4(a) and 4(b)). The permanent histology section (Figures 4(c) and 4(d)) and immunostains (Figure 4(f)) showed finding consistent with cardiac myxoma.

## 2. Discussion

Primary cardiac tumors are extremely rare with an estimated incidence rate of 0.1%. In certain studies, benign cardiac tumors are estimated to represent more than 90% of cardiac tumors [1].

Myxomas account for more than half of all benign cardiac tumors and most frequently appear in the left atrium (up to 75%) [2]. Other benign cardiac tumors include pericardial cysts (6.5%), papillomas (4%), fibromas (2.5%), rhabdomyosarcomas (2.5%), hematic cysts (1.5%), teratomas (1.5%), hemangiomas (0.8%), celotheliomas (0.8%), and lipomas (0.8%).

The most common malignant cardiac tumors are mesothelioma and myosarcoma (each 2.5%), followed by angiosarcoma (1.5%), fibrosarcoma (1.5%), and leiomyosarcoma (1.5%) [3]. The rate of incidence of metastatic cardiac tumors is not precisely known; however, one study reported 8% cardiac involvement in patients who died from metastatic cancer. [4]

Presentation of cardiac tumors, including myxomas, can be variable and mainly depends on the tumor location, size, and mobility. Asymptomatic cardiac myxoma cases have been reported in 10% in some studies [5]. Symptomatic cardiac myxomas can present with constitutional, obstructive, or embolic symptoms. Constitutional symptoms were reported in as high as 90% of the cases; however, these symptoms are nonspecific. Obstructive symptoms may manifest as progressive heart failure, syncope, valvular abnormalities variable with position, or sudden cardiac death. Arterial blood supply can also be affected by left atrial myxomas, with systemic emboli reported in 10-45% of cases; embolization to cerebral, coronary, renal, splenic, ocular, and cutaneous arteries have been reported [5].

In contrast to left atrial myxomas, right-sided atrial myxomas tend to be asymptomatic until relatively late in their development. One study demonstrated that right atrial myxomas are only symptomatic when 7 cm or greater, corresponding to around twice the size of when left atrial myxomas become symptomatic [6]. A right atrial tumor can obstruct the flow of blood through the tricuspid valve, leading to tricuspid stenosis and right heart failure. There is also a chance the tumor may break off and embolize to the lungs, causing pulmonary embolism [7], or cause systemic embolism if a patent foramen ovale or atrial septal defect is present. Villous surface of the mass increases the risk of embolization [7].

Physical exam findings are mainly auscultative. The pathognomonic “tumor plop” is a protodiastolic heart sound with low frequency and heard after the second heart sound. It can be confused with the third heart sound. An association between auscultative findings and symptomatic presentations has been reported [5].

The rarity of primary cardiac tumors, including intracardiac myxomas, makes them a challenge to diagnose. Many cardiac myxomas are found incidentally via imaging studies. Two-dimensional echocardiography is the gold-standard in diagnosis of intracardiac myxomas, with a transesophageal approach being superior to the transthoracic approach in its ability to visualize and define these tumors. Diagnosis can be adequately made with echocardiography alone by identifying the typical appearance of an atrial myxoma as a mobile mass attached by a visible stalk to the surface of the endocardium, commonly from the fossa ovalis [2]. Echocardiogram is also useful to assess hemodynamics and degree of obstruction. If this is not visible, typically, cardiac CT or MRI is required to better characterize the tumor and attempt to distinguish it from the appearance of mural thrombi. It also can help mapping coronary and cardiac anatomy for planning of a cardiothoracic surgery [8].

To our knowledge, there have been no documented cases of a right atrial myxoma concurrent with an endometrial adenocarcinoma. In the management of synchronous tumors, the most life-threatening condition should be treated first [9]. Another case of a left atrial myxoma and endometrial adenocarcinoma being managed concurrently has been reported in a 77-year-old female; surgeons elected to respect her pelvic mass to address her malignancy and left her asymptomatic cardiac tumor in place [10]. In our case, the patient underwent cardiac surgery to resect the mass, and the endometrial cancer was managed subsequently.

### 2.1. Follow-Up

The patient recovered well after the heart surgery. She underwent total laparoscopic hysterectomy and bilateral salpingo-oophorectomy for the endometrial cancer. Final pathology diagnosis was stage IIIC1 endometrioid endometrial adenocarcinoma. She was started on chemotherapy and continues to be followed. A follow-up TEE was significant for mild tricuspid regurgitation and showed a preserved ejection fraction of 60-65%.

## 3. Conclusion

Primary cardiac tumors are rare compared to metastatic tumors. These tumors can present with embolic or obstructive symptoms; it can also be asymptomatic even with larger sizes depending on its location. Multiple imaging modalities including echocardiography, cardiac CT, and MRI can be utilized to diagnose the tumors. Surgical resection is the treatment of choice for cardiac myxomas. Although no known available medical treatment for cardiac myxomas, it can still be used to manage tumor complications.

## Data Availability

The data used to support the findings of this study are restricted by the ethics board at Detroit Medical Center in order to protect patient privacy. Data might be provided for researchers who meet the institutional criteria for access to confidential data.

## Abbreviations

CD4: Cluster differentiation 4
TEE: Transesophageal echocardiogram
CT: Computerized tomography.

## Additional Points

*Learning Objectives.* (1) To be able to approach intracardiac masses and make a differential diagnosis. (2) To understand the possible different presentations of cardiac myxomas.
